# Microscale pressure measurements and optical coherence tomography reveal time-dependent biomechanical stages of ovulation in mice

**DOI:** 10.1016/j.isci.2025.114086

**Published:** 2025-11-22

**Authors:** Emily J. Zaniker-Gomez, Zihang Yan, Jing Yang, Darryl L. Russell, Hao Zhang, Sean X. Sun, Francesca E. Duncan

**Affiliations:** 1 Department of Obstetrics and Gynecology, Feinberg School of Medicine, Northwestern University, Chicago, IL 60611, USA; 2 Department of Biomedical Engineering, Northwestern University, Evanston, IL 60208, USA; 3 Department of Mechanical Engineering, The Johns Hopkins University, Baltimore, MD 21218, USA; 4 Institute of NanoBioTechnology (INBT), The Johns Hopkins University, Baltimore, MD 21218, USA; 5 Robinson Research Institute, School of Biomedicine, The University of Adelaide, Adelaide, SA, Australia

**Keywords:** medicine, veterinary medicine, pharmaceutical science, health profession, chemistry, physics, biological sciences

## Abstract

During ovulation, antral follicles undergo coordinated remodeling that results in egg release and corpora lutea formation, which are key processes for fertility and endocrine function. Using an *ex vivo* murine ovulation model integrated with time-lapse imaging, microscale pressure sensing, and optical coherence tomography (OCT), we characterized and quantified the sequence of mechanical events driving follicle rupture. We demonstrated that ovulation begins with a hyaluronan-dependent rise in intrafollicular pressure, followed by antral expansion and thinning of the follicle wall, which leads to elevated wall stress preceding rupture. Additionally, we characterized changes in ovulatory biomechanical dynamics associated with advanced reproductive age. By mapping the physical timeline of ovulation, this work establishes a framework for understanding the biomechanical regulation of egg release and provides insight into how age-related changes in follicular biomechanics may contribute to infertility.

## Highlights

• Ovulation is associated with an early increase in intrafollicular pressure
• Intrafollicular pressure dynamics during ovulation are hyaluronan-dependent
• Follicle wall thinning and elevated wall stress precede follicle rupture
• Reproductive aging disrupts follicle pressure dynamics and egg quality

## Graphical abstract

**Figure:**
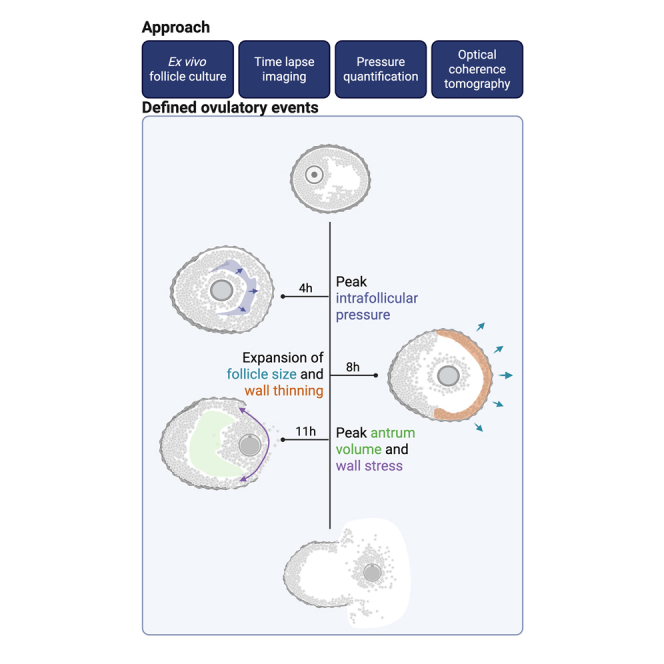

## Introduction

During ovulation, a mature antral follicle undergoes dramatic structural and molecular remodeling that culminates in the release of a fertilization-competent egg.^1^^,^^2^^,^^3^^,^^4^^,^^5^^,^^6^ Ovulation also supports the ovulatory cycle through the formation of a progesterone-producing corpus luteum.^6^^,^^7^^,^^8^ Therefore, ovulation is central to human fertility and endocrine function. Within ovulatory follicles there is a fluid-filled antral cavity that expands, leading to follicle rupture, which is necessary for successful ovulation.^2^^,^^9^^,^^10^^,^^11^^,^^12^ For decades, it was speculated that the fluid accumulation in the antrum coincides with an increase in cavity pressure that bursts the follicle wall.^13^ Studies to date are conflicting as to whether the ovulatory period is associated with an increase in intrafollicular pressure.^14^^,^^15^^,^^16^^,^^17^

A primary limitation in addressing this fundamental question has been the lack of technology to probe antral cavity dynamics and intrafollicular pressure precisely. Here, we combined an autonomous murine *ex vivo* ovulation model with time-lapse imaging, microscale pressure-sensing technology, and optical coherence tomography (OCT) to comprehensively study the follicle-inherent structural and biomechanical changes that underlie ovulation.^18^^,^^19^^,^^20^^,^^21^ Using these methods, we show that ovulation proceeds first with an early hyaluronan-dependent increase in intrafollicular pressure, followed by expansion of the antral cavity and follicle wall thinning, culminating in quantifiably elevated wall stress prior to rupture. We also demonstrate that intrafollicular pressure dynamics and follicle expansion during ovulation are disrupted with advanced reproductive age, which is associated with ovulatory dysfunction.^22^

Defining a timeline of ovulation from a biomechanical perspective provides essential insights into ovulation physiology. The technologies used in this study also establish a foundation for future mechanistic studies on the impact of elevated intrafollicular pressure on ovulation and fertility. Our study supports and expands upon prior investigations of intrafollicular pressure during ovulation and provides quantitative and mechanistic insights into the physiology of ovulation.

## Results

### Follicles expand and exhibit size-independent increased intrafollicular pressure during ovulation

**Figure 1:**
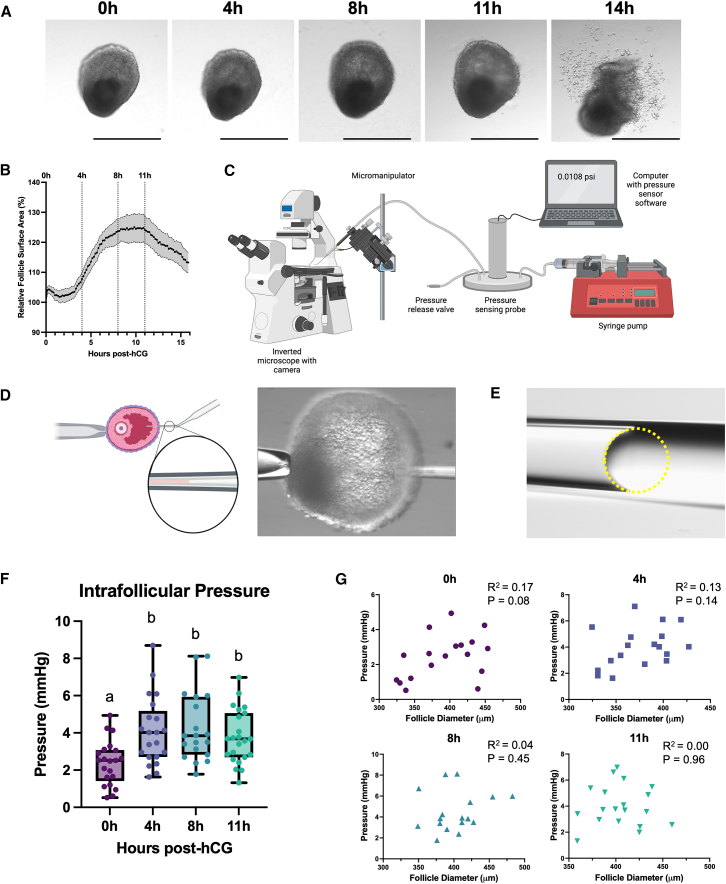
Follicles expand and exhibit size-independent increased intrafollicular pressure during ovulation (A) Representative images of a single follicle during ovulation at 0, 4, 8, 11, and 14 h after ovulation induction through exposure to hCG. Scale bars, 400 μm. (B) Follicle surface area, quantified using time-lapse imaging, expands in a sigmoidal pattern up until follicle rupture, after which it begins to contract. The growth curve is presented as the average ± SEM (shaded area), *n* = 6 follicles. (C) Schematic of microscale pressure measurement device, as described in Yang et al.^18^ (D) Schematic (left) and image (right) of an antral-stage follicle held in place with a holding pipette as the glass micropipette is inserted into the antral cavity. (E) Representative image of an oil-media interface overlaid with a yellow dashed circle fitted to the curvature. (F) Intrafollicular pressure increases from 0 to 4 h post-hCG and remains elevated through 11 h post-hCG. Values are plotted as a box-and-whisker plot displaying the mean, quartiles, and range. *n* = 19–23 follicles per time point. Data labeled a and b have statistically significant differences at a threshold of *p* < 0.05, one-way ANOVA. (G) There is no significant correlation between follicle diameter and pressure at any time point. Each point represents an individual follicle, and plots were fitted with a simple linear correlation. R^2^ and *p* values were reported for each correlation. *n* = 19–23 follicles per time point.

To quantify intrafollicular pressure dynamics during ovulation, we used a well-established *ex vivo* follicle culture and ovulation model that recapitulates key morphologic and molecular signatures of *in vivo* ovulation.^20^^,^^21^^,^^23^^,^^24^^,^^25^^,^^26^^,^^27^^,^^28^^,^^29^^,^^30^^,^^31^^,^^32^^,^^33^^,^^34^^,^^35^^,^^36^^,^^37^^,^^38^ Rupture and cumulus-oocyte-complex (COC) release is a regulated process that occurs in a gradual, stepwise fashion, which is recapitulated in our model system. This includes consistent expansion of the antral cavity, formation of a distinct rupture site, release of an expanded cumulus-oocyte complex, and formation of a progesterone-producing corpus luteum following ovulation.^20^^,^^21^^,^^23^^,^^24^^,^^25^^,^^26^^,^^27^^,^^28^^,^^29^^,^^30^^,^^31^^,^^32^^,^^33^^,^^34^^,^^35^^,^^36^^,^^37^^,^^38^ In this model, antral follicles grown *ex vivo* are induced to ovulate by treatment with human chorionic gonadotropin (hCG), an analog of luteinizing hormone (LH), which drives follicle rupture over the course of 12–14 h.^19^^,^^20^^,^^21^ We monitored the progression of ovulation by imaging follicles at 0, 4, 8, and 11 h after exposure to hCG (Figure 1A). These time points represent a pre-ovulatory state (0 h); two mid-ovulatory points (4 and 8 h), during which follicles are highly transcriptionally active and undergoing volumetric expansion; and a late ovulatory period (11 h) just prior to follicle rupture.^21^^,^^39^ To quantify changes in follicle size during ovulation, we used brightfield time-lapse imaging to monitor follicles every 10 min over the course of 14 h. From these images, we quantified the surface area of individual follicles and determined that follicles expand following a sigmoidal growth curve during ovulation (Figure 1B). The maximum expansion velocity occurs from 4 to 8 h post-hCG, with peak follicle surface area occurring between 8 and 11 h post-hCG (Figure 1B). After rupture occurs, the follicle wall contracts, and the surface area decreases (Figure 1B).

Intrafollicular pressure across ovulation was quantified using a microscale pressure-sensing system that functions by measuring curvature changes due to pressure-based displacement between fluid within a cavity of interest and an immiscible fluid (mineral oil) at the fluid-fluid interface (Figure 1C).^18^ This system has been used previously to measure pressure successfully in complex biological systems, including mammary gland organoids and mouse blastocysts.^18^ Pressure measurements were made in antral-stage follicles held stationary during insertion of the micropipette attached to the pressure-sensing probe (Figure 1D). The oil-follicular fluid interface was imaged prior to and after probe insertion into the antral cavity (Figure 1E). From these images, the cavity pressure was calculated using a derivation of the Young-Laplace principle.^18^ Intrafollicular pressure increased early during the ovulatory process, from 0 to 4 h post-hCG exposure, and this elevated pressure was sustained throughout the remainder of the ovulatory period (Figure 1F). The intrafollicular pressure remained constant between 4 and 11 h post-hCG (Figure 1F). There was no significant correlation between follicle diameter and pressure at any measured time point during ovulation, indicating that the increase in intrafollicular pressure is not directly correlated to an increase in size on an individual follicle basis (Figure 1G).

### Follicle expansion during ovulation is associated with asymmetric wall thinning, expansion of the antral cavity, and increased wall stress

**Figure 2:**
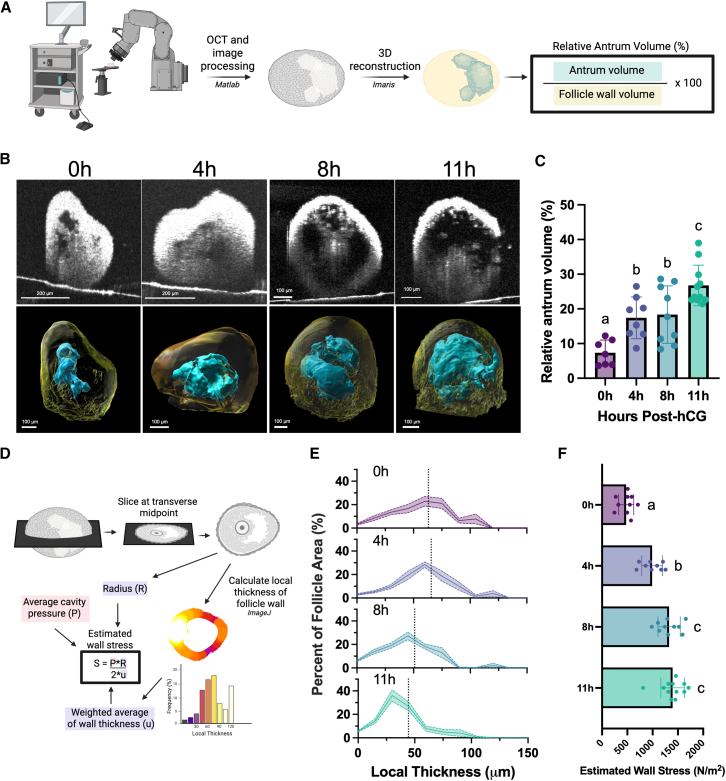
Follicle expansion during ovulation is associated with expansion of the antral cavity, wall thinning, and increased wall stress (A) Follicles were imaged using OCT, and images were processed and exported into Imaris for 3D reconstructions of the follicle wall and antral cavity. Relative antrum volume was calculated as the ratio of antrum volume to follicle wall volume. (B) Representative images of follicles at 0, 4, 8, and 11 h post-hCG are shown as a cross section from OCT (top) and a 3D reconstruction of the same follicle (bottom). The follicle volume is reconstructed in yellow, and the antral cavity is reconstructed in blue. Scale bars, 200 μm (top) and 100 μm (bottom). (C) Antrum volume increases from 0 to 4 h and again from 8 to 11 h post-hCG. *n* = 7–11 follicles per time point. Data labeled a, b, and c have statistically significant differences at a threshold of *p* < 0.05, one-way ANOVA. Error bars show SEM. (D) Schematic of measurements completed for wall thickness and wall stress. OCT images were sliced at the vertical midpoint, and the local thickness of the follicle wall was computed in ImageJ. The radius was also calculated and used to compute estimated wall stress using Laplace’s Law. (E) Distributions of wall thickness for follicles at each time point show a shift to a thinner average wall thickness. The weighted average of wall thickness is represented as a vertical dotted line. *n* = 9–12 follicles per time point. Shaded area shows SEM. (F) Estimated wall stress increases from 0 to 4 and 4 to 8 h post-hCG. *n* = 9–12 follicles per time point. Data labeled a, b, and c have statistically significant differences at a threshold of *p* < 0.05, one-way ANOVA. Error bars show SEM.

To further define changes in follicular architecture that occur during ovulation, we used OCT, an imaging technique developed for ophthalmologic applications that uses near-infrared light interference patterns to reconstruct 3D tissues.^40^ OCT provides an advantage over conventional high-resolution microscopy approaches, which require fluorescence and have limited depth penetration depending on laser power. Such features pose a challenge given the large size of antral follicles in the mouse, which are >400 μm.^41^ In contrast, OCT has a depth penetration of up to 3 mm and has been used to study *in vivo* ovulation dynamics and other reproductive tissues.^42^^,^^43^^,^^44^ Using OCT, we visualized and generated 3D reconstructions of both the follicle wall and antral cavities of isolated *ex vivo*-grown follicles across the time course of ovulation (Figures 2A and 2B). Using the reconstructions, we quantified the volume of the antral cavity (blue), which was normalized to the quantified volume of the entire follicle wall (yellow), to determine the proportion of the follicle volume made up by the antrum at each time point (Figure 2B). Antrum expansion increased steadily during ovulation and reached a maximum volume at 11 h post-ovulation induction (Figure 2C).

Follicle wall thinning is a necessary precursor for follicle rupture.^2^^,^^3^^,^^45^^,^^46^^,^^47^^,^^48^^,^^49^ To determine the timing and magnitude of follicle wall thinning during ovulation, we quantified the thickness of the follicle wall at the transverse midpoint of each follicle (Figures 2D and S1). This measurement quantifies the thickness of the follicle wall throughout the entire cross-section, which can be plotted as a distribution of wall thickness (Figures 2D and S1). At 0 and 4 h, the average follicle wall thickness was 63.1 and 65.6 μm, respectively, with a small proportion of the follicle wall that was >100 μm thick (Figure 2E). This suggests that prior to the onset of ovulation and during the early stages of ovulation, there is already asymmetry in the thickness of the follicle wall, likely in preparation for eventual rupture.^2^^,^^28^ At 8 and 11 h, there was a shift toward a smaller average wall thickness (50.9 and 44.4 μm, respectively), with a small proportion of the follicle wall retaining an increased thickness of around 80 μm (Figure 2E). These findings demonstrate that as the antral cavity expands between 4 and 11 h post-hCG, the follicle wall thins globally. At all time points, particular regions of the follicle wall exhibited increased thickness, suggesting that the follicle wall retains asymmetry even as it thins.

Wall stress, the force exerted on a cavity wall due to fluid pressure, is a known driver of structural and molecular remodeling in other cellular contexts.^50^^,^^51^^,^^52^^,^^53^^,^^54^ To determine how the changes in intrafollicular pressure, follicle size, and wall thickness we observed are related to stress exerted on the follicle wall, we combined follicle-specific measurements with average intrafollicular pressure measurements at each time point using Laplace’s Law (Figure 2D).^17^^,^^55^ This calculation incorporates the radius, average cavity pressure, and average wall thickness into an estimation of wall stress (Figure 2D). We used average intrafollicular pressure as an estimate for pressure at each time point, as we could not measure intrafollicular pressure in the same follicles imaged with OCT. Using the transverse cross-section of the OCT images for each follicle, we quantified the average follicle radius and the average follicle wall thickness, derived from a weighted average of the local thickness measurements (Figures 2D and 2E). The estimated wall stress increased throughout the ovulatory period and peaked at 8–11 h post-hCG (Figure 2F). This suggests that while pressure increases by 4 h post-hCG, further expansion of the antral cavity and thinning of the follicle wall are necessary for peak wall stress to be achieved, which may drive eventual rupture.

### Follicle expansion and increased pressure during ovulation are hyaluronan-dependent

**Figure 3:**
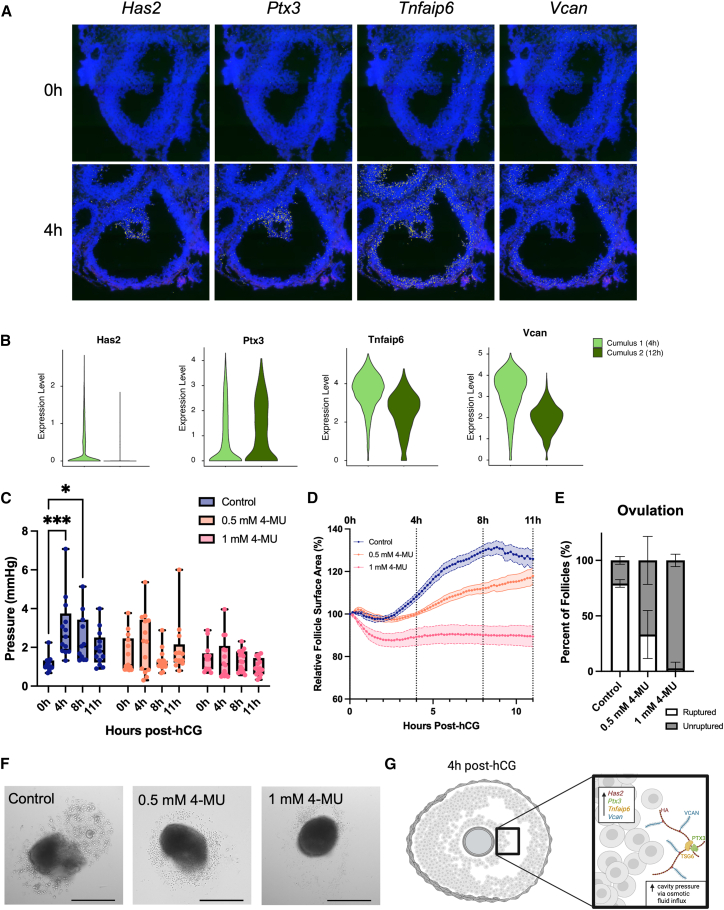
Follicle expansion and increased pressure during ovulation are HA-dependent (A) Genes related to HA matrix production and stability (*Has2*, *Ptx3*, *Tnfaip6*, and *Vcan*) are expressed in cumulus cells at 4 h post-hCG in a publicly available spatial transcriptomics dataset from Ruixu and Huang et al.^39^ Cell nuclei are stained in blue, and individual mRNA transcripts are visualized in yellow. (B) mRNA expression of *Has2*, *Ptx3*, *Tnfaip6*, and *Vcan* was detectable in early cumulus cells (4 h post-hCG) in the scRNAseq dataset from Ruixu and Huang et al.^39^ Expression of *Ptx3*, *Tnfaip6*, and *Vcan* was also detectable in late cumulus cells (12 h post-hCG). (C) Follicles treated with 0.5 and 1 mM of 4-MU do not exhibit an increase in pressure during ovulation. *n* = 11–13 follicles per time point per condition. ∗*p* < 0.05, ∗∗∗*p* < 0.001, two-way ANOVA. Values are plotted as a box-and-whisker plot displaying the mean, quartiles, and range. (D) 4-MU reduces follicle expansion during ovulation in a dose-dependent fashion. Data are presented as mean ± SEM (shaded area), *n* = 5–7 per condition. (E) 4-MU treatment causes a dose-dependent reduction in follicle rupture. Data are presented as mean ± SEM (error bars), *n* = 4 replicates, 15–20 follicles per condition in each replicate. (F) Representative images of control, 0.5 mM 4-MU, and 1 mM 4-MU-treated follicles at 14 h post-hCG. Scale bars, 400 μm. (G) Schematic of the proposed mechanism of HA-matrix mediated increase in antrum pressure through osmotic fluid influx.

To examine the molecular underpinnings of the establishment of intrafollicular pressure, we investigated the role of hyaluronic acid (HA), a glycosaminoglycan produced by the COC during ovulation, which is important for antral cavity expansion and ovulation.^5^^,^^9^^,^^56^ We proposed that production of HA, driven by hyaluronan synthase 2 (*Has2*) and matrix-stabilizing components—including versican (VCAN, *Vcan*), pentraxin 3 (PTX3, *Ptx3*), and tumor necrosis factor-inducible gene 6 protein (TSG6, *Tnfaip6*)—by cumulus cells in the first 4 h post-hCG, is a driver of increased intrafollicular pressure through osmotic fluid influx.^5^^,^^9^^,^^57^^,^^58^^,^^59^^,^^60^ To support this hypothesis, we evaluated expression of genes related to HA production and matrix stability during ovulation using a publicly available spatial transcriptomics and single-cell RNAseq (scRNAseq) dataset.^39^ We found that expression of *Has2*, *Ptx3*, *Tnfaip6*, and *Vcan* is enriched in cumulus cells of ovulatory follicles at 4 h post-hCG (Figure 3A). Consistent with previous reports, *Has2* expression was elevated in early cumulus cell clusters (4 h post-hCG), while *Ptx3*, *Tnfaip6*, and *Vcan* are expressed in both early (4 h) and late (12 h post-hCG) cumulus cells in the scRNAseq dataset (Figure 3B).^21^ These results support a potential role of HA in driving an intrafollicular pressure increase by demonstrating expression of key HA matrix-related genes during the early ovulatory period, which coincides with increasing intrafollicular pressure. To determine whether HA dynamics regulate intrafollicular pressure changes during ovulation, we treated follicles during ovulation induction with 4-methylumbelliferone (4-MU), a well-established inhibitor of HA synthesis in the context of the ovarian follicle.^41^^,^^60^^,^^61^ We compared intrafollicular pressure in untreated control follicles and those treated with 4-MU at 0, 4, 8, and 11 h post-hCG. 4-MU treatment blocked the normal increase in intrafollicular pressure, which was correlated with a dose-dependent decrease in follicle expansion (Figures 3C and 3D). Treatment with 4-MU also led to a dose-dependent decrease in follicle rupture, with the highest dose of 4-MU resulting in an incidence of follicle rupture of only 3% compared to 80% in the control group (Figures 3E and 3F). These results demonstrate that HA synthesis is required to regulate both follicular expansion and increased intrafollicular pressure during the early ovulatory process (Figure 3G).

### Reproductive aging is associated with follicle-intrinsic defects in ovulatory mechanics and outcomes

**Figure 4:**
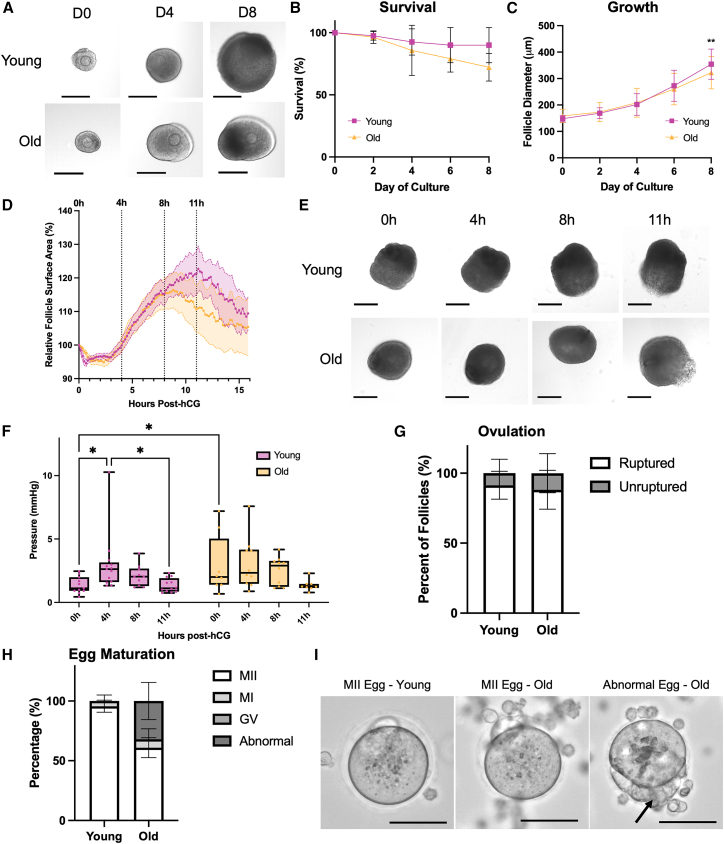
Reproductive aging is associated with follicle-inherent defects in follicle growth, ovulation mechanics, and egg quality (A) Representative images of follicles from reproductively young (6–12 weeks) and reproductively old (10–12 months) at 0, 4, and 8 days of *ex vivo* culture. Scale bars, 200 μm. (B) Follicles from reproductively old mice tended to have a lower survival rate by day 8 of culture. *n* = 3 replicates, 20 follicles per group in each replicate. Error bars show standard deviation. (C) Follicles from reproductively old mice grew to a significantly smaller terminal diameter by day 8 of culture. *n* = 3 replicates, 20 follicles per group in each replicate, with only surviving follicles quantified for growth. ∗∗*p* < 0.01, two-way ANOVA. Error bars show standard deviation. (D) Follicles from reproductively old mice exhibited a different pattern of expansion during ovulation, with a smaller and earlier peak size and early contraction. *n* = 6–7 per group, represented as mean ± SEM (shaded area). (E) Representative images of follicles from reproductively young and old mice at 0, 4, 8, and 11 h post-hCG. Scale bars, 200 μm. (F) Follicles from reproductively old mice did not have a significant increase in pressure from 0 to 4 h post-hCG and had a significantly higher intrafollicular pressure at 0 h post-hCG. *n* = 8–13 follicles per group per time point. ∗*p* < 0.0, two-way ANOVA. Values are plotted as a box-and-whisker plot displaying the mean, quartiles, and range. (G) The percentage of follicles that ruptured during ovulation did not differ between reproductively young and old mice. *n* = 5 replicates, 10–15 follicles per group in each replicate. Error bars show SEM. (H) Follicles from reproductively old mice showed a higher prevalence of abnormal oocytes collected after ovulation. Error bars show SEM. (I) Representative images of a metaphase II (MII) stage egg collected from a follicle from a reproductively young mouse, an MII stage egg collected from a follicle from a reproductively old mouse, and an abnormal egg collected from a follicle from a reproductively old mouse with clumped cumulus cells underneath the zona pellucida (arrow). Scale bars, 50 μm.

To determine whether ovulatory biomechanics and intrafollicular pressure are impacted in models of ovulatory dysfunction, we analyzed follicles from mice of advanced reproductive age, since aging is associated with higher rates of failed ovulation *in vivo* in mice.^22^^,^^23^^,^^62^ We isolated and cultured preantral follicles from reproductively young and old mice to the antral follicle stage (Figure 4A). Consistent with prior studies, follicles from reproductively old mice had a lower overall survival rate, and those follicles that did survive reached a smaller terminal diameter by day 8 of culture (Figures 4B and 4C).^23^^,^^62^ This suggests that there are follicle-inherent differences in follicular growth dynamics with age. When we examined follicle expansion during ovulation using time-lapse imaging, follicles from reproductively old mice exhibited an altered expansion pattern, with reduced magnitude and earlier timing of peak follicle expansion at around 8 h post-hCG (Figures 4D and 4E). In addition, follicles from reproductively old mice did not exhibit an increase in intrafollicular pressure during the ovulatory period and instead remained at a relatively consistent pressure, with no significant change throughout ovulation (Figure 4F). Notably, follicles from reproductively old mice exhibited a higher baseline intrafollicular pressure at 0 h post-hCG compared to young counterparts (1.36 ± 0.66 mmHg in young at 0 h vs. 2.88 ± 2.36 mmHg in old at 0 h; Figure 4F). Despite these differences in follicle growth and intrafollicular pressure, there was no difference in the ability of follicles to undergo rupture between groups when ovulation was induced with hCG (Figure 4G). Although follicle rupture was equivalent irrespective of age, follicles from reproductively old mice produced a higher proportion of abnormal eggs compared to young controls (Figure 4H). Many of these abnormal eggs exhibited accumulation of densely packed cumulus cells within the zona pellucida (Figure 4I). Taken together, these results suggest that there are follicle-inherent defects in follicle growth, ovulation biomechanics, and egg quality with advanced reproductive age, despite overall similar rates of *ex vivo* follicle rupture.

## Discussion

The relevance, and even the existence, of an increase in antral cavity pressure during ovulation has been a point of debate for nearly a century.^13^^,^^14^^,^^15^^,^^16^^,^^17^ While early studies found no significant changes in pressure during ovulation *in vivo*, the use of modern pressure-sensing technologies with higher resolution at a microscale has identified a gradual increase in intrafollicular pressure during *in vivo* ovulation.^13^^,^^14^^,^^15^^,^^16^^,^^17^ Our study is the first to assess intrafollicular pressure changes during ovulation in an isolated *ex vivo* system, which allows antral cavity dynamics to be quantified independently of the contribution of the broader ovarian microenvironment. Consistent with results from a previous *in vivo* study, we found that intrafollicular pressure increases during the ovulatory period.^17^ However, in contrast to this study, which reported a peak in intrafollicular pressure during the late ovulatory period, we observed an increase in pressure early (4 h post-hCG), which was then sustained up until 11 h post-hCG. This difference could be driven by variations in the organism, pressure-sensing methodology, or differences between *in vivo* and *ex vivo* ovulation. During *in vivo* ovulation, there are substantial changes to the follicle microvasculature that are thought to contribute to both antrum expansion and follicle rupture through increased vascular permeability and vasoconstriction of the follicle apex, respectively, which could translate to pressure that continues to mount *in vivo* during the late ovulatory period.^4^^,^^63^^,^^64^^,^^65^^,^^66^^,^^67^ Additionally, the presence of nearby immature and growing follicles could also influence the breakdown of the follicle wall.^68^ However, while these changes may contribute to ovulation dynamics *in vivo*, the ability of our assay to recapitulate follicle rupture in the absence of an intact vasculature suggests that vascular remodeling is not critical for the process of rupture itself.

We also used OCT to assess antral cavity and follicle wall dynamics during the ovulatory period.^42^^,^^43^^,^^44^ Combining our pressure results with measurements taken from OCT imaging, we were able to define a timeline of ovulation from a biomechanical perspective. Antral cavity pressure increases early in ovulation (4 h post-hCG), prior to significant expansion of the antral cavity, follicle size, or thinning of the follicle wall. From 4 to 8 h post-hCG, intrafollicular pressure is maintained, there is an increase in follicle size and antrum expansion, and there is more global thinning of the follicle wall. From 8 to 11 h post-hCG, pressure is maintained at an elevated level, and there is a further increase in antrum volume and thinning of the follicle wall. The estimated follicle wall stress increases gradually throughout ovulation and peaks during the late ovulatory period (8 and 11 h post-hCG). We performed each technique (pressure measurements and OCT) on an individual follicle only once at a single time point to avoid confounding effects of repeated removal from the incubator, insertion of the micropipette, or exposure to the lasers from OCT. Despite not being able to perform parallel measurements in the same follicle, we nevertheless documented consistent changes during the time course of ovulation, indicating that these dynamics are robust. Other methods, such as time-lapse multiphoton microscopy, which allow for continuous monitoring and imaging of a single follicle, may have higher sensitivity to monitor changes in the follicle wall or antral cavity geometry.^41^ Additionally, fluorescence-based imaging systems allow for identification of cumulus cells or antral fluid components that are not clearly delineated with OCT imaging.^41^ The development of future technologies that could monitor these changes at higher temporal resolution would further refine the timing and magnitude of these key changes. Additionally, smooth muscle-like contractions of the follicle have a documented role in ovulation, both *in vivo* and in *ex vivo* models.^16^^,^^41^^,^^66^^,^^67^^,^^69^^,^^70^^,^^71^^,^^72^ Integration of imaging modalities at high temporal resolution, or pharmacologic studies using smooth muscle inhibitors combined with pressure measurements, will help elucidate any role that contractility of the follicle wall plays in the maintenance of elevated intrafollicular pressure during late ovulation. Additionally, future studies modulating enzymes such as matrix metalloproteinases (MMPs) and tissue inhibitors of MMPs, which are important for follicle wall degradation and eventual rupture, will help elucidate the relationship between thinning of the follicle wall and intrafollicular pressure during ovulation.

A particularly intriguing result from our study is that pressure increases early during ovulation and remains consistent thereafter. While the antral cavity remains intact until follicle rupture, the overall follicle and the antrum both increase in size throughout this period, which may lead to maintenance of increased pressure after an initial surge. This could play several roles in supporting ovulation and egg maturation. First, it could promote migration and expulsion of the COC across the antral cavity, which is necessary for appropriate release of the egg into the oviduct or fallopian tube.^2^^,^^4^ It could also play a role in egg maturation itself, potentially by promoting retraction of transzonal projections (TZPs) between cumulus cells and the maturing egg, which is a necessary step for resumption of meiosis. This potential role is supported by work demonstrating the positive impact of elevated pressure on egg maturation *ex vivo* and the presence of mechanosensitive machinery on COCs that could be responsive to changing pressure dynamics.^73^^,^^74^^,^^75^^,^^76^^,^^77^^,^^78^^,^^79^ In addition, directly tracking intrafollicular pressure at particular time points during ovulation in relation to egg maturation and fertilization outcomes could establish a link between pressure and egg quality. Future studies could identify follicles with different magnitudes of intrafollicular pressure changes during ovulation to determine whether such metrics are predictive of ovulation or egg quality outcomes. Finally, increasing intrafollicular pressure may play a role in driving follicle wall thinning, as changes in fluid force exposure have a known impact on the structural and transcriptomic properties of cells.^52^^,^^53^^,^^54^^,^^80^ Understanding the role that antral cavity biomechanics play in driving follicle rupture could, therefore, help identify factors that contribute to poor egg quality and anovulatory infertility.

We also identified HA as a critical mediator of intrafollicular pressure dynamics during ovulation. We determined this by treating follicles with 4-MU to block synthesis of HA, a glycosaminoglycan that drives antrum expansion and is known to be essential for follicle rupture.^9^^,^^41^^,^^61^^,^^81^ HA has been previously implicated as a mediator of intraocular pressure increases and is a known regulator of osmotic gradients in many biological systems.^82^^,^^83^^,^^84^^,^^85^^,^^86^ In addition, altered HA production and function have been implicated in disorders associated with ovulatory dysfunction, including reproductive aging and polycystic ovarian syndrome.^87^^,^^88^^,^^89^^,^^90^^,^^91^ We found that follicles treated with 4-MU had no increase in intrafollicular pressure and exhibited a dose-dependent reduction in follicle expansion during ovulation. This suggests that HA plays a key role in driving the increase in intrafollicular pressure during ovulation, likely through mediating expansion of the antral cavity. Interestingly, in our system, pressure increases early in ovulation (4 h post-hCG), which is before peak production of HA in the antral cavity.^92^ However, previously published studies of HA production by cumulus cells and our own analysis of existing spatial transcriptomics and scRNAseq data provide evidence of early production of HA and the associated matrix early in ovulation, which drives antral cavity expansion through an osmotic gradient, promoting increased intrafollicular pressure.^21^^,^^39^^,^^92^ Ovulation dysfunction associated with impaired HA production, therefore, may also be driven by altered intrafollicular pressure dynamics.^89^^,^^91^ Future studies will extend this work to assess the role of active fluid channels that are known to contribute to antrum expansion, including aquaporin 9, on ovulation and antral cavity dynamics, in order to further elucidate the link between the expanding antral cavity and intrafollicular pressure.^9^^,^^10^

We also used aging as a physiologic model of ovulatory dysfunction.^22^ Reproductive aging in mice is characterized by decreased follicle growth and survival, impaired follicle rupture, reduced HA levels, reduced egg quality, and fibroinflammatory changes to the stroma.^22^^,^^23^^,^^60^^,^^62^^,^^93^^,^^94^^,^^95^^,^^96^^,^^97^^,^^98^^,^^99^^,^^100^^,^^101^^,^^102^ We found that follicle expansion and intrafollicular pressure dynamics were altered with age. Reproductively old follicles have a reduced peak expansion, an earlier time to the peak, and do not exhibit an increase in intrafollicular pressure. While total hyaluronan levels decrease in the ovary and in follicular fluid with age, this likely does not directly contribute to the establishment of baseline intrafollicular pressure due to low overall expression of Has2 at the preovulatory stage.^60^^,^^90^ Further investigation is necessary to define the role of altered Has2 signaling on intrafollicular pressure changes with aging as ovulation progresses. Instead, other factors, including differences in follicle size or follicle wall architecture, may contribute to the differences in baseline pressure observed with age. We also observed increased variability in the pressure measurements of aged follicles, consistent with increased variability seen in other parameters of reproductive aging, including gene expression of eggs and stiffness of the extrafollicular environment.^90^^,^^103^ This is consistent with the concept that aging is not binary but rather a continuum and that not all follicles from reproductively aged mice will be perturbed to the same degree. Despite these changes, follicles from reproductively old mice are able to rupture and ovulate similarly to young controls. Thus, these results suggest that the block in ovulation with age observed *in vivo* is more likely due to the age-dependent increase in fibrosis and stiffness of the ovarian stroma.^22^^,^^90^^,^^98^^,^^104^^,^^105^^,^^106^^,^^107^ In fact, acute treatment with antifibrotic agents can rescue the ovulatory defects.^101^^,^^104^^,^^108^ Nevertheless, it is possible that the reduced follicle expansion and altered intrafollicular pressure dynamics we observed with age may predispose these follicles to exhibit ovulatory defects when combined with an additional challenge of the aging microenvironment, which becomes fibrotic and stiff. Future studies incorporating a stiff microenvironment into *ex vivo* culture conditions, or additional *in vivo* studies of ovulation biomechanics with age, will be important to further clarify this point.^109^

Our study defines ovulation temporally from a biomechanical perspective, demonstrating that ovulation is characterized by an early increase in intrafollicular pressure, which is followed by antrum expansion, follicle wall thinning, and increased wall stress. The observation that pressure increases early in ovulation and plateaus suggests that follicle rupture is not exclusively driven by mounting pressure reaching a particular threshold that causes the follicle wall to rupture. Instead, increased intrafollicular pressure, in combination with follicle wall thinning, drives increasing wall stress, which supports eventual rupture, egg maturation, and COC translocation across the antrum.^2^^,^^4^^,^^52^^,^^53^^,^^54^^,^^73^^,^^74^^,^^75^^,^^76^^,^^77^^,^^78^^,^^79^ Understanding the role that antral cavity biomechanics play in follicular rupture could, therefore, help identify factors that contribute to poor egg quality and anovulatory infertility. Ultimately, the technology we describe in this study will serve as a foundation for addressing many of these mechanistic questions regarding the role of pressure and antral cavity expansion during normal and impaired ovulation.

### Limitations of the study

It is important to note that, while *ex vivo* cultured follicles recapitulate key structural and molecular features of *in vivo* ovulation, there may still be features of ovulation physiology that are not fully replicated in our system, particularly with respect to the role of the extrafollicular environment and vasculature.^21^^,^^28^ Despite this potential limitation, our system allows for investigation of ovulation dynamics in a highly controlled environment using multimodal technologies that would not be possible in an *in vivo* approach. Additionally, our use of enzymatic digestion during follicle isolation may impact the integrity of the follicle basement membrane, which could result in differences between *in vivo* and *ex vivo* pressure measurements; further experiments are warranted to directly compare *ex vivo* models of ovulation. Nevertheless, follicles exposed to enzymes during isolation are still capable of producing an intact antrum and undergoing follicular rupture in response to ovulatory cues. In addition, with all pressure-sensing technologies that involve puncturing the fluid-filled cavity, there is a possible risk of fluid leakage through the small puncture site, which may impact the magnitude of pressure measurements. However, this is highly unlikely, as we used a small-sized micropipette (1–3 μm), visualized follicle morphology during pressure measurements, and ensured viability by assessing follicle architecture and complete follicle rupture following measurements. Moreover, following this process, follicles remained viable and were fully able to undergo the process of ovulation. Finally, there are limitations in the translatability of our results to the physiology of human ovulation. While many aspects of ovulatory physiology are conserved between mammalian species, the use of mouse models rather than human ovarian tissue is a limitation.^1^ Future studies of ovulation biomechanics in large mammalian models, such as bovine or nonhuman primates, may reveal additional insights that are not feasible to study in human models.

## Resource availability

### Lead contact

Requests for further information and resources should be directed to and will be fulfilled by the lead contact, Francesca E. Duncan (f-duncan@northwestern.edu).

### Materials availability

This study did not generate new reagents.

### Data and code availability

• All data reported in this paper will be shared by the corresponding author upon request.
• This paper does not report original code.
• Any additional information required to reanalyze the data reported in this paper is available from the lead contact upon request.

## Acknowledgments

We would like to thank Britt Goods, Caroline Kratka, Ruixu Huang, and Rhea Sharma for their assistance with the spatial transcriptomics and scRNAseq data. This work was supported by the Eunice Kennedy Shriver National Institute of Child Health and Human Development of the 10.13039/100000002National Institutes of Health under award no. 1F30HD113284-01A1 (E.Z.) and by the Gates Foundation grant INV-003385 (F.E.D.). Under the grant conditions of the Gates Foundation, a Creative Commons Attribution 4.0 Generic License has already been assigned to the author-accepted manuscript version that may arise from this submission.

## Author contributions

E.J.Z.-G. wrote the manuscript and contributed to experimental design, data collection, and data analysis. Z.Y. contributed to experimental design and data collection. J.Y., D.L.R., H.Z., S.X.S., and F.E.D. contributed to experimental design, data interpretation, manuscript writing, and approvals. All authors participated in discussions regarding this manuscript and approved its final submission.

## Declaration of interests

The authors declare no competing interests.

## STAR★Methods

### Key resources table

**Table:** 

REAGENT or RESOURCE	SOURCE	IDENTIFIER
**Chemicals, peptides, and recombinant proteins**		

Leibovitz’s L-15	Thermo Fisher Scientific	Cat. No. 11415064
αMEM Glutamax	Thermo Fisher Scientific	Cat. No. 32561037
DMEM/F-12 Glutamax	Thermo Fisher Scientific	Cat. No. 10565018
Fetal Bovine Serum (FBS)	Peak Serum Inc.	Product Code PS-FB1
Bovine Serum Albumin (BSA)	Sigma-Aldrich	Cat. No. A9647-500G
Recombinant Follicle Stimulating Hormone (rFSH), Gonal-F	Merck Group	N/A
Insulin Transferrin Sodium Selenite (ITS)	Sigma-Aldrich	Cat. No. I3146
Penicillin-Streptomycin	Thermo Fisher Scientific	Cat. No. 15140122
Deoxyribonuclease I (DNAse I)	Sigma-Aldrich	Cat. No. D4263
Liberase DH	Sigma-Aldrich	Cat. No. 05401054001
Human Chorionic Gonadotropin (hCG)	Sigma-Aldrich	Cat. No. C1063
Recombinant Epidermal Growth Factor (mEGF)	Sigma-Aldrich	Cat. No. SRP3196
4-Methylumbelliferone sodium salt (4-MU)	Sigma-Aldrich	Cat. No. M1508
Alginic Acid Sodium Salt	Sigma-Aldrich	Cat. No. 71238
Alginate Lyase	Sigma-Aldrich	Cat. No. A1603

**Experimental models: Organisms/strains**		

CD1	Envigo	N/A

**Software and algorithms**		

ImageJ/Fiji Version 2.9.0	NIH, USA	https://imagej.net/ij/notes.html
GraphPad Prism, Version 10 for Max OS X	GraphPad Software, La Jolla California USA	https://www.graphpad.com
Imaris Version 10.2	Oxford Instruments	N/A
Microsoft Excel, Version 16	Microsoft, Inc.	N/A
MERSCOPE Visualizer Software	Vizgen	https://vizgen.com/vizualizer-software/

**Other**		

Dino-Lite Digital Camera	Dunwell Tech, Inc.	N/A
CellBox Shipper Flight	Cellbox Solutions GmbH	N/A
Microscale Pressure Sensor	Johns Hopkins University	N/A

### Experimental model and study participant details

#### Animals

Female CD-1 mice were used for all experiments (Envigo, Indianapolis, IN). For experiments where prepubertal mice (PND 14-16) were used for follicle isolation and culture, adult female CD-1 mice were purchased with 7-day-old female pups. In these experiments, prepubertal mice were used to maximize follicle yield for an appropriately powered sample size. For experiments studying reproductive aging, reproductively young (6–12-week-old) and reproductively old (10–12-month-old) mice were used and were housed in groups of up to 5 females per cage. Reproductively old mice were purchased as retired breeders. All mice were held in polypropylene cages at the Northwestern University animal facility for a minimum of 7 days prior to use to ensure acclimation. Mice were provided with food and water *ad libitum* and were kept in a temperature-, humidity-, and light-controlled barrier facility with 14-hour light/10-hour dark cycles. All animal procedures were approved by the Northwestern University Institutional Animal Care and Use Committees (IACUC), and we have complied with all relevant ethical regulations for animal use (protocol number IS00016799).

#### Follicle isolation and encapsulated *ex vivo* follicle growth assay

For all experiments in this study, follicles were isolated from ovaries using an enzymatic digestion method. *Ex vivo* follicle culture using an enzymatic approach was performed according to an established protocol to maximize follicle yield while maintaining key morphologic and molecular features of ovulation.^20^^,^^21^^,^^23^^,^^24^^,^^25^^,^^26^^,^^27^^,^^28^^,^^29^^,^^30^^,^^31^^,^^32^^,^^33^^,^^34^^,^^35^^,^^36^^,^^37^^,^^38^ *In vivo* approaches or antral follicle isolation techniques are more limited in terms of yield and feasibility, particularly in the context of advanced reproductive age. Beyond feasibility, approaches using isolated antral follicles require culture in small droplets that exert forces from the surface tension that may alter antral cavity biomechanics. Additionally, we wanted to exclude the role of the follicle microenvironment and vasculature in our study and instead focus on follicle intrinsic factors. Ovaries were removed the ovarian bursa and cut into 4-8 uniform pieces using a scalpel. Ovary pieces were incubated in L15 media (Thermo Fisher Scientific, Waltham, MA) with 1% penicillin-streptomycin (Thermo Fisher Scientific), 25 μg/ml liberase DH (Sigma-Aldrich, St. Louis, MO), and 200 μg/ml deoxyribonuclease I (DNAse I; Sigma-Aldrich) for 20 minutes at 37°C on a plate shaker. After 20 minutes, the enzymatic solution was quenched with 10% fetal bovine serum (FBS; Peak Serum Inc., Wellington, CO) and the ovary pieces were manually broken down by repeated pipetting using a wide bore P1000 pipette. Multilayer secondary stage follicles (150–180 μm diameter) with a uniform, intact morphology were selected and encapsulated in a 0.5% (w/v) alginate hydrogel (Sigma-Aldrich), which maintains the 3D structure of follicles throughout culture. Follicles are selected based on strict morphologic criteria requiring well-defined oocyte borders with even, translucent layers of granulosa cells. Based on this strict criteria, unhealthy follicles are removed and may represent follicles that have already started to undergo atresia. In addition, follicles are rescued from FSH-dependent competition that occurs *in vivo*. The selection of high-quality follicles and overall survival of around 80% of follicles in culture represents aspects of *in vivo* follicle selection during folliculogenesis.^19^ Individual follicles were encapsulated within 5 μl alginate hydrogel drops that were crosslinked in a 50 mM CaCl2 (Thermo Fisher Scientific) and 140 mM NaCl (Thermo Fisher Scientific) solution for 2 min. Individual multi-layer secondary follicles within beads were cultured in individual wells of a 96-well plate. Follicles were cultured in growth media, which consists of 50% αMEM Glutamax (Thermo Fisher Scientific) and 50% DMEM/F-12 Glutamax (Thermo Fisher Scientific) supplemented with 3 mg/ml bovine serum albumin (BSA; Sigma-Aldrich), 10 mIU/ml recombinant follicle stimulating hormone (rFSH; Gonal-F, Merck & Co., Rahway, NJ), 1 mg/ml bovine fetuin (Sigma-Aldrich), and 5 μg/ml insulin-transferrin-sodium selenite (ITS, Sigma-Aldrich). Follicles were cultured for 8 days at 37°C in a humidified environment of 5% CO2 in air with half of the growth media replaced every 2 days.

### Method details

#### *Ex vivo* ovulation

Cultured follicles were used for an *ex vivo* ovulation assay. Fully grown antral follicles, identified by diameter ≥ 400 μm and presence of a visible antral cavity, were selected for ovulation. Selected follicles were removed from alginate by incubating in L15 media containing 1% FBS (Peak Serum Inc.) and 10 IU/ml alginate lyase from flavobacterium multivorum (Sigma-Aldrich) at 37°C for 20 min. After removal from alginate, follicles were then washed in L15 media (Thermo Fisher Scientific) containing 1% FBS (Peak Serum Inc.) to remove any partially digested alginate residue. Unencapsulated follicles were then transferred to 100 μl of maturation media containing 50% αMEM Glutamax (Thermo Fisher Scientific) and 50% F-12 Glutamax (Thermo Fisher Scientific) supplemented with 10%FBS (Peak Serum Inc.), 1.5 IU/ml human chorionic gonadotropin (hCG; Sigma-Aldrich), 10 ng/ml epidermal growth factor (EGF; Sigma-Aldrich), and 10 mIU/ml rFSH (Merck & Co.) in individual wells of a 96-well plate. Follicles were incubated in a humidified environment of 5% CO2 in air for up to 16 hours. After 14-16 hours of exposure to maturation media, follicles were visually assessed to determine rupture status. Rupture was defined as a visible break in the integrity of the follicle wall with the presence of cumulus cells or a cumulus-oocyte-complex outside of the follicle wall. For aging studies, oocytes were manually collected and image following ovulation to determine maturation status.

#### Time lapse imaging

Follicles selected for time lapse analysis of ovulation were placed in an incubator containing an RK-10A mount holding a Dino-Lite Edge AF4915ZTL microscope with N3C-R light focuser (Dino-Lite Digital microscopes, Torrance, CA, USA). The microscope was focused, and contrast was adjusted to provide maximum resolution at approximately 75x magnification. Follicles were imaged every 10 minutes for 16 hours during ovulation.

#### Microscale pressure sensor

Experimental set up is shown in Figure 1C, as previously described.^18^ This device is capable of measuring hydraulic pressures at lengths scales of 10 microns. A custom pressure chamber was connected to a syringe pump (NE-1000, New Era Pump Systems, Inc., 50 ml syringe, B-D Company) to regulate the holding pressure within the chamber. This was connected to a pressure transducer (PX409, Omega Engineering, Inc.), which connects to software that monitors pressure within the chamber. The pressure sensing device was then connected to a micromanipulator set up (Eppendorf Transfer Man NK2) attached to an inverted microscope (Nikon Eclipse TE300) with imaging capabilities. Glass micropipettes were manually pulled using an automatic pipette puller (Sutter Instruments), after which the pipette was bent at a 20-35 degree angle over the flame of a Bunsen burner. The tip of the pipette was opened to a < 10 μm diameter and then was manually loaded with mineral oil. The pipette was then installed on the pipette holder for pressure measurements. Prior to pressure measurements, calibration measurements were taken by adjusting the syringe pump to a range of pressure levels and imaging the oil-media interface, which is used to derive the surface tension of the media (Figure S2).^18^ Individual follicles were placed one at a time in 2 mL drop of holding media, which consists of L15 media containing 1% FBS and 0.5% penicillin-streptomycin, on the inverted lid of a 50 mm petri dish. The follicle was fixed in place using a holding pipette (Vitrolife, Sweden) and imaged. Next, the oil-media interface of the pipette was imaged prior to insertion and the gauge pressure was noted. Then, the pipette tip was gently inserted into the antral cavity of the follicle. At this time, the oil-media interface was imaged again and the gauge pressure was noted. This was repeated for each follicle at each time point. After measurements, follicles were returned to the incubator to be imaged at 14-16 hours post-hCG to assess for rupture. Follicles that failed to rupture, except for 4-MU treated follicles, were excluded from analysis.

#### Optical coherence tomography

Follicles were placed in a 35mm petri dish with 3 mL of maturation media for imaging. A custom-built vis-OCT system was employed to image the follicles. The system has a theoretical axial resolution of 1.3 μm in tissue and a theoretical lateral resolution of 9.4 μm. Image acquisition focused on identifying distinct follicle structures, quantifying their volume and wall thickness, and monitoring the dynamic changes at different time points. Each scan covered a 1.58 × 1,58 mm^2^ field of view, consisting of 512 A-lines and 512 B-scans, repeated twice per B-scan and three times per volume. Prior to and after imaging, follicles were held in a CellBox Shipper Flight (CellBox Solutions, Hamburg, Germany) portable incubator at 37°C and 5% CO2.

#### 4-MU treatment

A subset of follicles were exposed to 4-MU (Sigma-Aldrich, M1508). 4-MU was resuspended in embryo-grade water (Sigma-Aldrich, W1503) and then mixed with maturation media at final concentrations of 0.5 and 1 mM. Control media contained maturation media mixed with the same volume of embryo grade water. Follicles treated with 4-MU were used for time lapse imaging and pressure quantification experiments.

#### Multiplexed error-robust fluorescence *in situ* hybridization (MERSCOPE) spatial transcriptomics and single-cell RNA-Seq (scRNAseq) analysis

Spatial transcriptomic and scRNAseq data derived from a publicly available dataset was used with permission. MERSCOPE images, processed as previously described in the original publication, were extracted using Visgen’s MERSCOPE visualizer software.^39^ Expression of *Has2*, *Ptx3*, *Tnfaip6*, and *Vcan* were visualized in ovarian sections collected at 0h and 4h post-hCG. Representative images from individual follicles at each time point with visible cumulus cells were selected. The same representative follicle was shown for each of the selected genes. Ovarian sections at the 12-hour time point did not contain COCs that were visible within antral follicles, and thus they were uninformative for our purposes and not included. Instead, gene expression was evaluated using our scRNAseq dataset that includes a late cumulus cell cluster present at only the 12-hour time point given the unique transcriptomic profile of cumulus cells late in ovulation. To evaluate expression of this set of genes in the scRNAseq dataset, we examined expression of *Has2*, *Ptx3*, *Tnfaip6*, and *Vcan* in the cumulus 1 (early cumulus cells present at 4h post-hCG) and cumulus 2 (late cumulus cells present at 12h post-hCG) clusters. Gene expression was represented as a violin plot with expression levels of each gene in each of the two cumulus cell clusters.

### Quantification and statistical analysis

#### Time lapse quantification

Time lapse images were imported into ImageJ for analysis. Time lapse images from individual follicles were saved into z-stacks with background removed. The threshold of each image was adjusted to capture the surface area of the follicle in each image. The area within the threshold of each image was calculated and plotted to represent change in surface area over time across the 16-hour period. Time points of interest (0, 4, 8, 11h) were added to graphs manually. Results were plotted as mean ± standard error of the mean (SEM), with shaded area representing the SEM.

#### Pressure quantification

Cavity pressure was quantified as previously described.^18^ Prior to the start of pressure measurements, a calibration experiment was performed in follicle holding media, which consists of L15 media containing 1% FBS and 0.5% penicillin-streptomycin, on the inverted lid of a 50 mm petri dish. The calibration curve was created by adjusting the syringe pump to hold the pressure chamber at a variety of pressures. At each pressure reading, the oil-media interface was imaged, and the radius of curvature was quantified in Fiji. The radius of curvature was plotted with gauge pressure and a linear regression was fitted to the plot. The slope of the linear regression represents the surface tension of the media. To quantify cavity pressure, the radius of curvature was quantified using ImageJ for the oil-media interface before and after insertion into the cavity. Using the surface tension, the radius of curvature before and after insertion, and the gauge pressure before and after insertion, the cavity pressure can be derived.^18^

#### OCT quantification

Reconstructed volumes were registered, averaged, and flattened to improve the signal-to-noise ratio. After processing, images were imported into Imaris 10.2.0 (Oxford Instruments) for 3D image analysis. 3D surfaces were generated from the follicle wall and the antral cavity using built in Imaris 3D surface classification tools. These surfaces were used to calculate the antral cavity volume, which was normalized to the directly calculated follicle wall volume.

To calculate the local wall thickness, a cross-sectional image was taken at the transverse midpoint of each individual follicle (Figure S1). The image was then uploaded into ImageJ, and the area occupied by the cumulus-oocyte-complex and any background from fluid based on 3D-image analysis in Imaris was removed. Cellular content was distinguished from fluid based on the signal brightness in the OCT images where cellular material was bright and fluid was dark. The COC was manually identified based on separation from the follicle wall and reduced pixel intensity relative to cells of the follicle wall. The follicle wall thickness was then analyzed using the ImageJ plugin “Local Thickness,” and the output was plotted as a histogram of thickness around the circumference of the cross section and normalized to the follicle size. For the quantification of estimated wall stress, the same cross-sectional images were used. Wall stress was calculated using the Law of Laplace (stress = [pressure ∗ radius] / [2∗ wall thickness]). Pressure was derived from the average pressure measurement at each time point. The radius was quantified on an individual follicle basis from the cross-sectional image. Average wall thickness was quantified using a weighted average of local thickness measurements.

#### Statistical analysis

Statistical analysis was completed using GraphPad Prism 10 (GraphPad Software Inc., San Diego, CA, USA). Data in figures is represented as either the mean ± standard deviation, mean ± standard error of the mean, or a box and whisker plot with the full range, as specified in figure legends. When comparing between multiple groups, a two-way ANOVA with Tukey correction for multiple comparisons was used to assess statistical significance between groups. To assess the relationship between follicle diameter and intrafollicular pressure, simple linear correlations were completed for each individual time point. A threshold of p-value < 0.05 was considered statistically significant. Sample sizes are noted in the figure legend for each dataset.
