# Supplementary material for: Microscale pressure measurements and optical coherence tomography reveal time-dependent biomechanical stages of ovulation in mice
Source: iScience. 2025 Nov 22;28(12):114086. doi: 10.1016/j.isci.2025.114086 (PMC12744267; doi:10.1016/j.isci.2025.114086)
Supplement: Document S1. Figures S1 and S2 [file mmc1.pdf]

iScience, Volume *28*

Supplemental information

Microscale pressure measurements and optical coherence tomography reveal time-dependent biomechanical stages of ovulation in mice

Emily J. Zaniker-Gomez, Zihang Yan, Jing Yang, Darryl L. Russell, Hao Zhang, Sean X. Sun, and Francesca E. Duncan

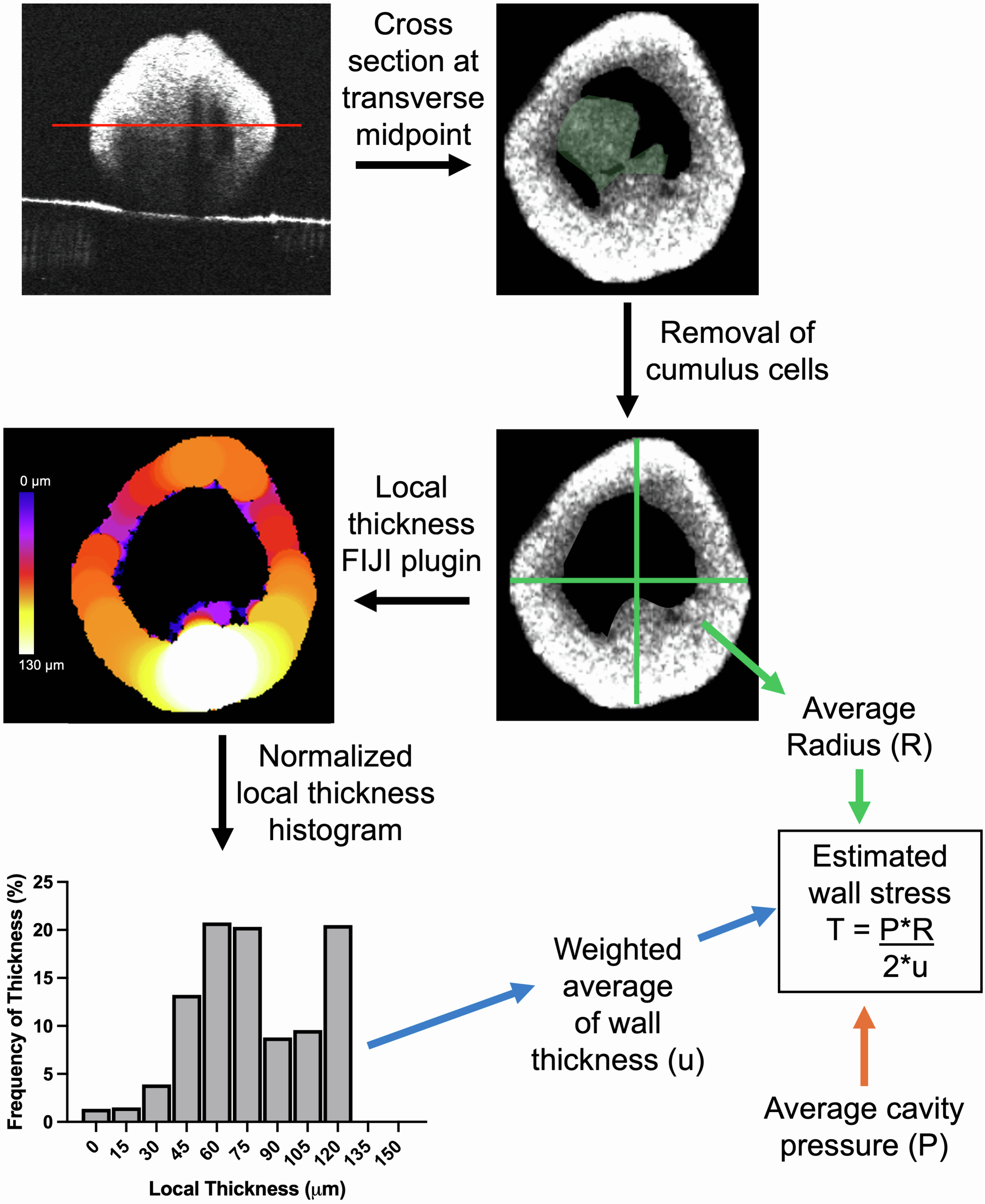

Supplemental Figure 1. Local thickness measurement strategy.
Representative image of a follicle cross-section processed in the local thickness measurement pipeline.

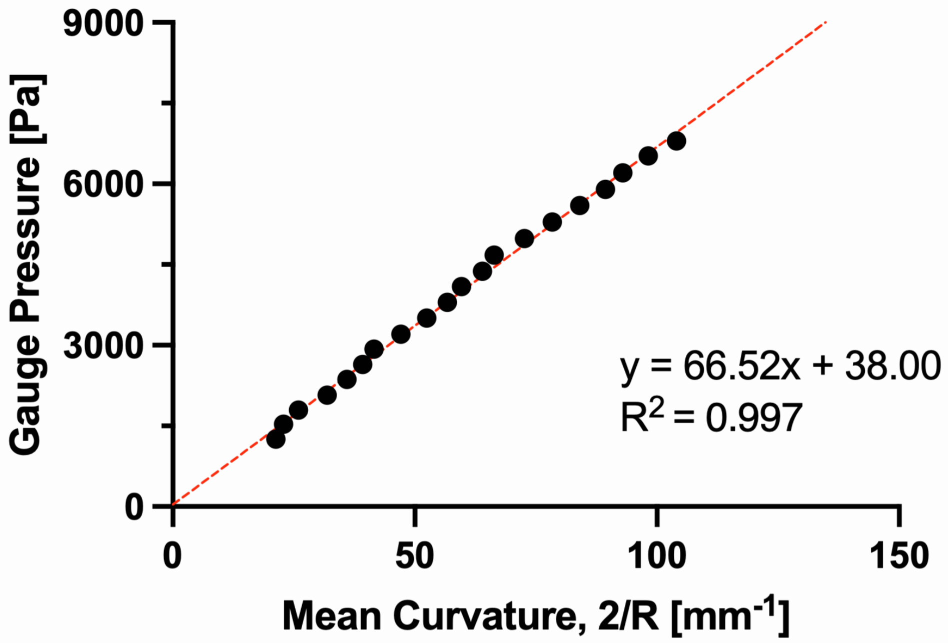

Supplemental Figure 2. Representative calibration curve.
Calibration curve acquired before each replicate of pressure measurements. The slope of the calibration curve represents the surface tension of the media.
